# The Role of Bacterial, Dentinal, Salivary, and Neutrophil Degradative Activity in Caries Pathogenesis

**DOI:** 10.3390/dj11090217

**Published:** 2023-09-15

**Authors:** Yuval Peled, Cameron A. Stewart, Michael Glogauer, Yoav Finer

**Affiliations:** 1 Faculty of Dentistry, University of Toronto, Toronto, ON M5G 1G6, Canada; yuval.peled@mail.utoronto.ca (Y.P.); cameron.stewart@mail.utoronto.ca (C.A.S.); michael.glogauer@dentistry.utoronto.ca (M.G.); 2 Institute of Biomedical Engineering, University of Toronto, Toronto, ON M5S 3E2, Canada; 3 Department of Dental Oncology, Maxillofacial and Ocular Prosthetics, Princess Margaret Cancer Centre, Toronto, ON M5G 2M9, Canada

**Keywords:** degradative activity, neutrophils, primary caries, secondary caries, restoration failure, resin-based restorations, collagen type-1

## Abstract

Until recently, it was widely accepted that bacteria participate in caries pathogenesis mainly through carbohydrate fermentation and acid production, which promote the dissolution of tooth components. Neutrophils, on the other hand, were considered white blood cells with no role in caries pathogenesis. Nevertheless, current literature suggests that both bacteria and neutrophils, among other factors, possess direct degradative activity towards both dentinal collagen type-1 and/or methacrylate resin-based restoratives and adhesives, the most common dental restoratives. Neutrophils are abundant leukocytes in the gingival sulcus, where they can readily reach adjacent tooth roots or gingival and cervical restorations and execute their degradative activity. In this review, we present the latest literature evidence for bacterial, dentinal, salivary, and neutrophil degradative action that may induce primary caries, secondary caries, and restoration failure.

## 1. Introduction

Dental caries, also known as “cavities” or “tooth decay,” is one of the most common chronic diseases worldwide and the leading cause of oral pain and tooth loss [1]. The World Health Organization (WHO) reports that up to 90% of school-aged children worldwide suffer from caries, mainly in developing countries [2]. In the USA, children lose approximately 51 million school hours due to oral illnesses such as caries each year [3]. As for adults in the United States, 9 out of 10 suffer from carious lesions, and 1 out of 5 have untreated tooth decay. Worldwide, however, 1 out of 3 adults have untreated caries [4].

In the 9th Edition of the Glossary of Prosthodontic Terms, caries is defined as a disease involving the destruction of the tooth caused by acid-producing, cariogenic bacteria [5]. The main cariogenic species are Mutans streptococci (*Streptococcus mutans* (*S. mutans*) and *Streptococcus sobrinus*), which are gram-positive facultative anaerobic cocci [6]. Initially, early colonizing microbes adhere to the tooth’s hard non-exfoliating tissues through the pellicles found on the tooth’s surface [7]. This is an essential step in bacterial colonization in the supragingival biofilm found above the gum line [6,7]. Then, bacterial species such as Mutans streptococci use the carbohydrates consumed in the diet to produce extracellular polymers such as glucans and fructans that form the biofilm matrix, a well-organized organism community [8]. As the biofilm matures, a pathogenic shift can occur when bacterial inhabitants ferment these carbohydrates to produce acids, such as lactic acid. These acids can lower the pH to acidic levels, which can induce demineralization of the tooth (removal of minerals from the tooth’s enamel, dentin, and cementum) [7,8,9]. Defensive elements such as salivary flow, buffer systems, fluoride use, and a non-cariogenic diet can prevent and reverse the demineralization process [7,10]. However, when bacterial acid production is too high, the pH can drop to a critical level, and demineralization of the tooth occurs. Eventually, this process may lead to tooth cavitation (tooth decay, caries, or carious lesions) [1,6,7,8,9,10]. 

Other vital factors in caries pathogenesis are endogenous host enzymes. Salivary and dentinal enzymes are known to contribute to dentin degradation [11,12,13] as well as the breakdown of resin-based composites (RBC) [14,15,16,17]. More recent studies have shown that human neutrophils could be key players in the caries mechanism [18,19]. These immune system cells are abundant inhabitants of periodontal tissues [20], and through the gingival sulcus, they reach the oral cavity [18,19]. There, they interact intimately with different tooth components, such as tooth dentin, cementum, RBC, the restoration-tooth interface, and different bacterial species. When activated, these leukocytes produce degradative activity and contribute to different tooth-restoration content degradation [18,19]. For this narrative review, we gathered the most recent data on bacterial and host degradative activity towards tooth dentin and resin-based restoratives and their contribution to caries and restoration failure. 

## 2. Methods

A comprehensive search of the most relevant literature was conducted. Data collected in this review was gathered from the PubMed database using the keywords “bacterial degradation”, “dentinal degradation”, “neutrophils degradation”, “salivary degradation”, “resin restorations”, “resin adhesives”, “tooth dentin”, and “dentin collagen”. We selected the most up-to-date publications examining the degradation of tooth dentin and resin-based monomers, restorations, and adhesives by bacterial, dentinal, salivary, and neutrophil activity. Studies that focused solely on indirect degradation (by cell recruitment and chemotaxis) were excluded from this review.

## 3. Caries Classifications

### 3.1. Primary Caries: Coronal and Root Caries

The tooth cavitation process can be initiated either in the crown portion of the tooth or in the tooth’s roots. The crown is covered with enamel and comprises dentin and a pulp chamber. The tooth roots are covered with cementum and mostly comprise dentin and pulp canals. Coronal caries usually begins with bacterial accumulation on pitted or smooth enamel surfaces. The enamel prisms comprise a mechanical barrier that is difficult to infiltrate, thus lengthening the cavitation process [9,21]. Furthermore, enamel contains a high inorganic content (90%) as opposed to dentin and cementum, which are less mineralized (70% and 40–50%, respectively) [21,22]. The dentin also lacks a prismatic enamel structure, containing a less organized structure of dentinal tubules with an organic component of mostly collagen type-1. The hollow structure of the tubule walls permits bacterial adhesion and invasion of these tubules [22]. All the above enables the biofilm to dissolve dentin with higher critical pH values (less acidic) compared with enamel at 6–6.8 and 5.4, respectively [21,22]. Another coronal caries feature is the creation of a protected anaerobic environment after bacterial penetration of the dentin-enamel junction (DEJ). This environment enables the biofilm to accumulate uninterruptedly in the less mineralized dentin tissue, shielded by the enamel layer [9]. 

In root caries biofilms, the main bacterial species are Mutans streptococci, Lactobacillus, and Actinomyces [21]. These highly acidogenic (acid-producing) and aciduric (acid-tolerant) bacterial species produce acids, such as lactic acid, which may lower pH values from 4.9 to 5.6, which is lower than the critical pH for both enamel and dentin [22]. This results in the dissolution of the dentin and cementum tissues and softening of the lesion surface at an earlier stage of the carious process, as compared with coronal caries [9,21]. Despite the rather high critical pH value of the dentin, root caries lesions located apically to the DEJ are usually up to only 1 mm deep. This finding could be attributed to the slow biofilm degradation rate and the lesion’s location, which can be exposed to proper hygiene control and fluoride toothpaste [19]. These measures could elevate pH levels and remineralize the cavitated surface, leading to an arrested carious lesion [19,22].

### 3.2. Secondary (Recurrent) Caries

Another form of the carious process is recurrent or secondary caries, which is defined as a carious lesion limited to the margins of a current restoration (dental filling) [23,24,25,26], with up to 90% of these lesions found in the gingival margins of class II (Proximal) and V (Cervical) dental restorations [25]. These margins are in constant contact with the sub-gingival bacterial plaque, saliva, gingival crevicular fluid (GCF), and different immune system cells such as neutrophils, exposing them to potential degradative activity [18,19,25]. These secondary caries lesions are also the most reported cause of re-restoration of teeth, conducted due to the failure of the previous restoration. This process includes the repair (when possible) or replacement of an existing damaged restoration, regardless of the kind of restorative material used [27]. These lesions appear mostly in caries-susceptible patients. These individuals often have a biofilm-welcoming environment caused by cariogenic dietary habits and impaired oral hygiene, which promote plaque formation. The carious lesions will occur especially in interproximal areas, which are relatively difficult to clean [25]. Another contributing factor is applying a dental restoration to a tooth with existing residual caries [24]. G.V. Black coined the term “Extension for prevention” in 1891 in cavitated teeth preparation and concluded that infected and affected dentin should be excavated completely as a part of tooth preparation [24]. This method was eventually replaced with a more preservative approach of minimally invasive dentistry, where affected non-carious dentin is maintained while infected dentin is fully removed. Notwithstanding, keeping soft demineralized dentin with residual caries at the tooth-restoration interface might compromise the restoration’s bond strength to the tooth structure, leading to secondary caries [25,26,28]. An additional etiologic cause is the presence of a primary carious lesion in adjacent regions of the treated tooth. This lesion could appear after applying the restoration; however, most primary carious lesions are reportedly present when the restoration is performed but go unnoticed. In advanced stages, the primary carious lesion can expand and potentially damage the restoration, resulting in secondary caries [23]. 

## 4. Bacterial, Dentinal, and Salivary Dentin Degradative Activity

### 4.1. Bacterial Degradative Activity towards Dentin

As stated above, cariogenic bacterial species initiate carious cavitation by acid generation that lowers the pH to a level that demineralizes dentinal collagen [1,6,7,8,9,10]. Nevertheless, bacteria exhibit enzymatic activity that may directly degrade dentinal collagen once it has been exposed to acid [29,30].

*Enterococcus faecalis* (*E. faecalis*) and *Micrococcus luteus* (*M. luteus*) are gram-positive bacteria associated with endodontic infection and root canal treatment failure. Marashdeh et al. found that when incubated with dentinal collagen, these bacteria produce highly active protease-like or matrix metalloproteinase (MMP)-like activity, which degrades collagen type-1. This activity could contribute to carious cavitation by removing material and exposing additional mineralized tissue, using digested collagen as a nutrient source, and by compromising the ability of restorative components to adhere to the infected dentin [29]. Huang et al. discovered that although *S. mutans* are widely known for their ability to dissolve tooth enamel and dentin through acid production [10,21,30], they also possess direct degradative activities towards dentinal collagen [30]. Their study showed that through the generation of intracellular and extracellular proteolytic activity, this bacterium can degrade both collagen type-1 and demineralized human dentinal collagen. This activity is growth-phase dependent, where late-growth *S. mutans* exhibit more degenerative activity than freshly incubated *S. mutans*. This activity could be attributed to the late-growth phase’s ability to generate selective proteases and intracellular enzymes in higher numbers, which can efficiently execute collagen degradation [30]. *E. faecalis*, *M. luteus*, and *S. mutans* can all generate dentinal collagen degradation, which, consequently, promotes carious lesion cavitation [29,30].

### 4.2. Dentinal Endogenous Degradative Activity towards Dentin

In the carious lesion process, cariogenic bacteria ferment carbohydrates and produce acids that dissolve dentin. These acidic conditions initiated by cariogenic bacteria activate endogenous salivary and dentinal enzymes such as MMPs and cysteine cathepsins. At neutral pH values, they are inactive pro-enzymes (zymogens) that participate in the breakdown of collagen under low pH conditions. There are 28 known types of MMPs that can be divided into six groups: gelatinase (MMP 2, 9), collagenase (MMP 1, 8, 13), stromelysin (MMP 3, 10, 11), matrilysins (MMP 7, 26), membrane type MMPs (MMP 14, 15, 16, 17, 24, 25), and other MMPs [12]. MMP enzymes found in dentin have an important role in dentinogenesis and dentin remodeling. The initial bacterial enzymatic activity generates lower pH values that demineralize the dentin and expose dentinal collagen type-1. As stated above, this low pH also activates previously latent dentinal and salivary proteases, suggesting that bacterial biofilm is responsible for initiating dentinal destruction in the earlier stages of the root carious lesion, both by acid production that demineralizes dentin [10,21,30], and by direct dentin degradation [29,30]. Once dentin demineralization begins, and pH values are low enough to activate the zymogens, collagen breakdown is attributed to dentinal, salivary, bacterial, and neutrophil degradative activity [13,18,19,29,30,31,32,33,34]. Nevertheless, it has been reported that the bacterial contribution to dentinal degradation is much higher than that of dentinal and salivary degradative activity [13,21,29,30]. 

The predominant MMPs found in the dentin compartments are gelatinases (MMP 2 and MMP 9), collagenases (MMP 8 and MMP 13), stromelysin (MMP), and membrane-type (MMP 14) [12,13,30,31,32]. The gelatinase MMP 2 is the main MMP in intact dentin and is known to play an important role in dentinogenesis. This enzyme is found in mineralized and demineralized human dentin matrices, with the latter indicating involvement in dentin extracellular matrix (ECM) degradation in carious lesions [13]. Furthermore, the gelatinase activity in carious dentin is higher than that of sound dentin when extracted from different dentin layers. This increase is confirmed by significantly higher MMP 2 gene expression in odontoblasts near carious dentin, leading to increased MMP 2 levels and subsequent dentinal degradation. [13,35,36]. Another dentinal enzyme, MMP 3, a stromelysin, has degradative potential towards dentin ECM components, as it is isolated from demineralized dentin in its active form. This stromelysin can cleave dentinal proteoglycans and non-collagenous proteins (NCPs) such as dentin sialoprotein, bone sialoprotein, and osteopontin when active [13,37].

In addition to MMPs, dentinal cysteine cathepsins are proteases that play important roles in carious processes. Studies have shown that compared with sound dentin immunostaining, cathepsin B demonstrated higher immunostaining in the dentinal tubules of demineralized carious dentin, with increasing depth (closer to pulp tissues). This finding is important for younger patients, as their dentin tubules are wider and more numerous, allowing a higher influx of pulp-derived cysteine cathepsins via higher volumes of tubular fluids. One proposed cathepsin dentin matrix degradation mechanism involves activating latent MMPs by cathepsin B and K [13,21,38]. 

### 4.3. Salivary Degradative Activity towards Dentin

Another potential contributor to dentin degradation and caries may be saliva-derived host enzymes. Salivary levels of collagenase MMP 8 are reportedly increased in subjects with cavitated carious lesions than in patients with no caries. The salivary MMP 8 and MMP 9 can originate from salivary glands, GCF [33,34], and neutrophils [18,19]. These proteases are hypothesized to encounter carious lesions on the outer surface of the dentin rather than in deeper lesions where they can degrade the dentinal matrix [33,34]. 

Tissue inhibitor of matrix metalloproteinase-1 (TIMP-1) is an inhibitor of MMP 8, also found in saliva and GCF, and is upregulated in carious lesions. The MMP 8/TIMP-1 ratio was used to reflect periodontal disease status and proteolytic activity, where a higher value ratio indicates an imbalance between the two [34,39]. At a high ratio, high levels of MMP 8 combined with low levels of TIMP-1 can indicate dysregulation, resulting in increased dentinal collagen type-1 degradation [34].

Salivary cysteine cathepsin, mainly cysteine cathepsin B, as well as MMPs, have been found in high numbers with increased activity in carious lesions [38,40]. Another report showed that salivary cysteine cathepsin B did not show a statistically significant difference between enzymatic activities at different lesion depths. By contrast, dentinal cysteine cathepsin B increases its activity in deeper carious lesions, especially in exposed pulp involvement. The report also reconfirmed that salivary MMPs present higher activity in active carious lesions, with mildly higher MMP activity shown in deeper carious lesions [34].

Although dentinal and salivary dentin collagen degradative ability is measurable [22,31,32,33,34], the bacterial MMP-like activity is around 50 times more potent, as shown in the Marashdeh et al. study [29]. This finding further reinforces the above assumption that the bacterial contribution to dentinal collagenolytic activity in caries is more significant than that of salivary and dentinal degradative activity [22,29]. 

## 5. Immune System Degradative Activity

### 5.1. Neutrophils

Neutrophils are bone marrow-derived leukocytes. These cells are key players in the innate immune system and comprise 50–70% of leukocyte circulation as the body’s first line of defence. Additionally, their production occupies up to 60% of the bone marrow space [41]. When encountering pathogens, these cells can perform phagocytosis (engulf microbes), execute degranulation, generate reactive oxidative species (ROS) that can kill pathogens, and create neutrophil extracellular traps (NETs). When sensing invading micro-organisms, neutrophils create NETs, which are uncondensed DNA materials comprising a network structure. This form contains bactericidal agents, serine proteases, and neutrophil elastases (NE) that can eradicate harmful bacteria [41,42,43,44].

#### 5.1.1. Neutrophils’ Degradative Activity in Periodontitis

Periodontitis is an inflammatory disease involving the destruction of the periodontal attachment apparatus [45,46,47]. *Porphyromonas gingivalis* (*P. gingivalis*) can induce an inflammatory response modulation in the host, reinforcing the destruction of periodontal tissues by the immune system [48,49]. Reports have shown that *P. gingivalis* may prevent neutrophils from harming pathogens while maintaining their pro-inflammatory actions [50]. When dysregulated, these neutrophils can directly degrade periodontal tissues by producing enzymes such as MMP 8 and MMP 9 [51,52,53]. 

#### 5.1.2. Neutrophils’ Degradative Activity towards Dentin

Recent discoveries have linked dental caries to the immune system cells, specifically neutrophils. It has been found that neutrophils can degrade tooth dentin and RBC, leading to root caries and secondary caries [18,19]. Their proximity to the cervical third of the tooth and the gingival or cervical restoration margins in the subgingival regions enables neutrophils to contribute to the carious process [18,19]. 

**Figure 1:**
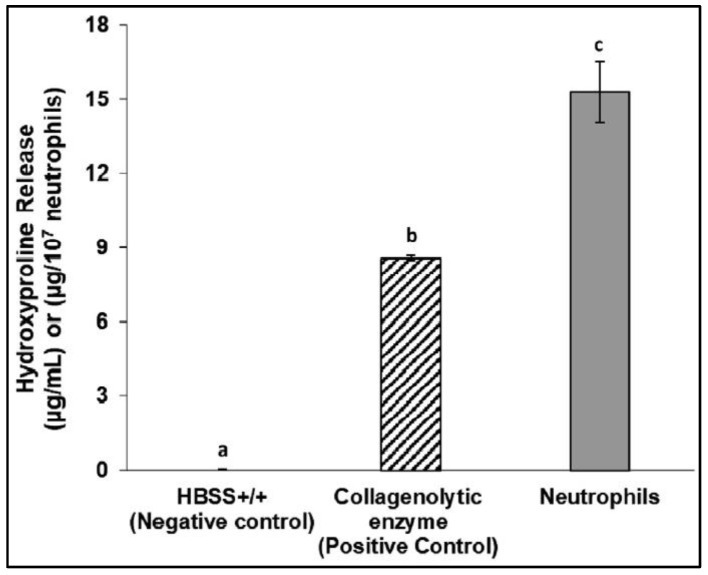
Hydroxyproline release after incubation (24 h, 37°C) of demineralized collagen samples with HBSS alone (negative control), collagenolytic enzymes (positive control), and neutrophils with HBSS media. Differences between neutrophil incubation and negative and positive control incubation can be observed after 24 h (Figure by Gitalis et al., 2019 [18]). Different letters represent statistically different values (*p* < 0.05).

**Figure 2:**
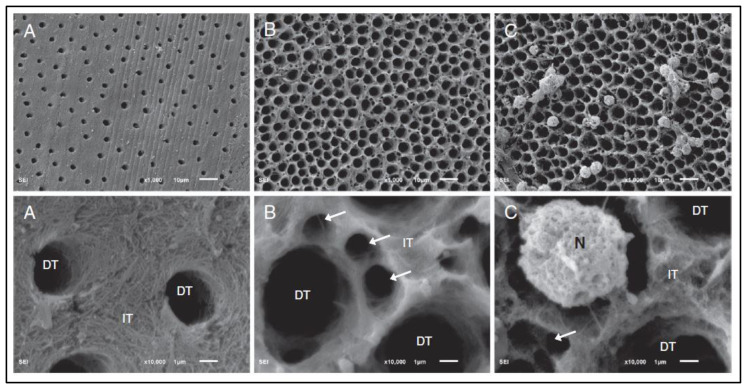
Scanning electron microscopy (SEM) image (top row shows ×1000 magnification and bottom row shows ×10,000 magnification) of collagen tubules and demineralized dentin tubules (DT). Sample A was incubated with HBSS (24 h, 37 °C), presenting undamaged dentin tubules and intratubular dentin (IT). Sample B was incubated with collagenolytic enzymes and saline (24 h, 37 °C), featuring degraded intratubular dentin and deteriorated fibrillar collagen (arrows). Sample C was incubated with neutrophils (N) and saline (24 h, 37 °C), displaying degraded intratubular dentin and deteriorated fibrillar collagen (arrow) (Figure by Gitalis et al., 2019 [18]).

Gitalis et al. investigated the effects of neutrophils on pre-demineralized collagen, which showed an increase in hydroxyproline, a degradation by-product of collagen, as compared with collagenolytic enzymes as the positive control and Hanks’ Balanced Salt solution (HBSS) alone as the negative control (Figure 1) [18]. When observing demineralized dentin specimens under scanning electron microscopy, incubating samples with neutrophils produced comparable results to collagenolytic enzymes, namely the loss of fibrillar collagen and degradation of intratubular dentin. Samples incubated with media alone had intact fibrillar collagen as well as undamaged intratubular dentin (Figure 2) [18].

Furthermore, neutrophils can reportedly degrade collagen type-1 mainly through gelatinase MMP 2 and MMP 9 activities than collagenase MMP 1 and MMP 8 activities, which were considerably less effective [18]. Since tooth dentin organic component comprises 90% collagen type-1 [21], this finding can shed light on the effect of neutrophils’ degradative activity on caries formation and restoration failure in human dentin [18]. MMP 8, a collagenase, is known to cleave collagen type-1, and MMP 9, a gelatinase, can break down denatured collagen [12,19]. 

Also, cathepsin G can activate the zymogen of MMPs [54], thus indirectly inducing dentin degeneration by stimulating salivary, dentinal, and neutrophil MMPs [19]. Another enzyme supporting MMPs’ function is neutrophil gelatinase-associated lipocalin (NGAL). This protein forms a stable complex with MMP 9 and supports its destructive activity by protecting the gelatinase from degradation [19,55]. Clinically, this enzymatic complex can prolong MMP 9 degradative activity towards tooth dentin in the oral cavity [19]. 

**Figure 3:**
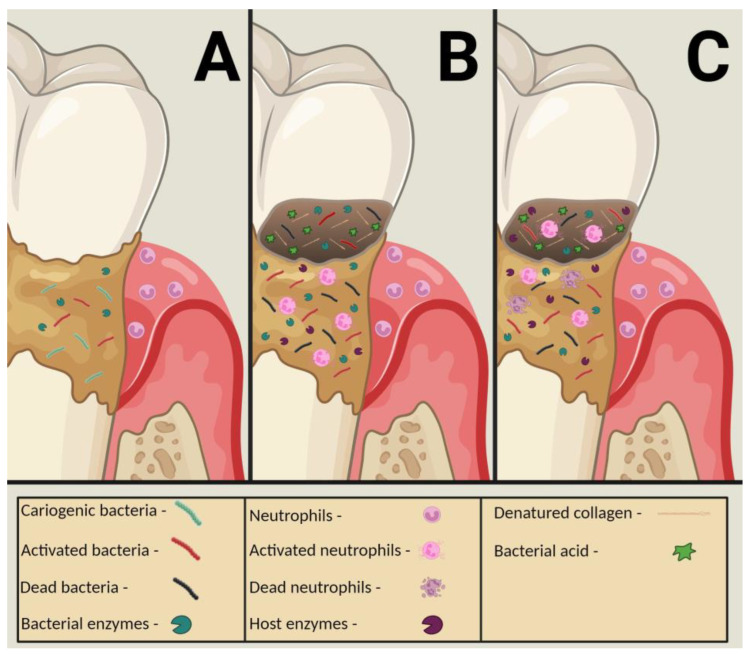
Illustration of a root/cervical carious lesion. (**A**)—Initial bacterial biofilm colonization of the tooth’s cervical portion, with bacterial enzyme release by activated bacteria. Neutrophils are in the adjacent periodontal sulcus. (**B**)—Advanced bacterial biofilm colonization of the tooth’s cervical portion. Carious cavitation of the tooth with demineralized collagen fibrils initiated by bacterial enzyme release and acid production by activated bacteria, with some bacterial apoptosis. Activated neutrophils migrate to the biofilm and emit neutrophil enzymes. Salivary enzymes are activated. (**C**)—Advanced bacterial biofilm colonization of the tooth’s cervical portion. Carious cavitation of the tooth with demineralized collagen fibrils initiated by bacterial enzyme release and acid production by activated bacteria, with some bacterial apoptosis. Activated neutrophils migrate to the carious lesion, with some neutrophil apoptosis. Neutrophil, salivary, and dentinal enzymes are activated and promote further collagen degradation.

In the caries process involving dentin, neutrophils can degrade demineralized rather than intact dentin [18,19], supporting the hypothesis that bacterial degradation and acid production precede endogenous degradative activity (Figure 3) [21,31]. Therefore, cariogenic bacteria are essential contributors to the carious lesion process [21,30,31]. They initiate collagen demineralization via acid production, provide low pH conditions to activate host zymogens [8,13], and produce the most impactful breakdown of dentinal collagen compared to host-derived degradation [30].

In addition to their degradative activity towards dentin, bacterial and host enzymes can degrade resin-based restoratives, thus contributing to secondary caries and restoration failure.

## 6. Resin-based Composites

### 6.1. RBC Chemistry and Composition

**Figure 4:**
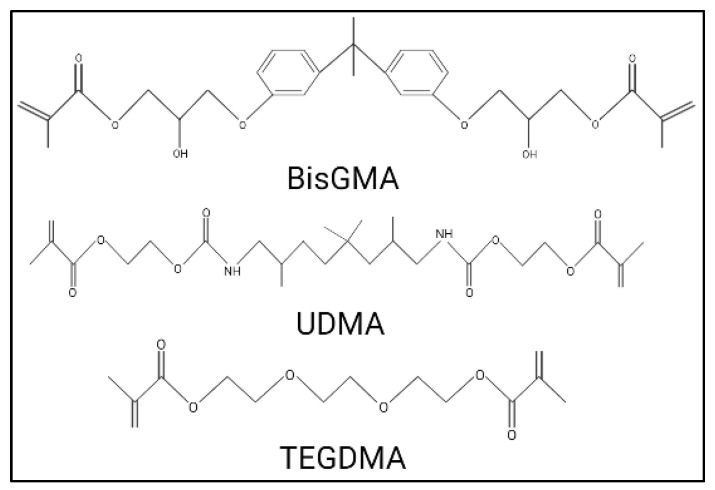
Chemical structures of common resin composite monomers.

Resin-based composites are tooth-coloured restorations that are currently the most popular dental restorative material [56]. RBCs contain methacrylate-based polymers, and glass or ceramic fillers [57,58]. They also contain dimethacrylate molecules that can adjust the cross-linked polymerization reaction of the matrix monomers and a silane coupling agent that links the fillers to the polymer matrix [57,58]. The RBC setting is attributed to a chemical reaction creating cross-linking between the methacrylate monomers. The most common RBC cross-linking monomers are 2,2-Bis [4-(2-hydroxy-3-methacryloxypropoxy) phenyl] propane (bisGMA), urethane dimethacrylate (UDMA), and triethylene glycol dimethacrylate (TEGDMA) (Figure 4). Dimensional shrinkage may accompany this reaction, imposing significant stress on the tooth-restoration bond [57,58,59].

### 6.2. The Adhesive System

Adhesive systems maintain a constant, mechanically durable, and adequately sealed bond between an RBC and the tooth [60]. It is more challenging to achieve sufficient restoration adhesion to dentin than to enamel due to the high organic content and smear layer created after cavity preparation [60,61,62]. Another challenge is the constant moisture provided by dentinal tubular fluids, which imposes a significant obstacle to adhesion as some adhesive systems are ineffective in moist environments. Other bonding materials might be compromised if applied to over-wet or over-dried dentin [63,64]. Adhesive systems can generally be divided into two subtypes: (1) Total-Etch (TE), also known as etch-and-rinse, and (2) Self-Etch (SE) systems. TE systems require the application of phosphoric acid (usually 30–40% concentration) followed by rinsing prior to applying the primer and adhesive. By contrast, SE systems contain acidic monomers and do not require a rinsing phase [57,65].

### 6.3. The Hybrid Layer

The hybrid layer is the resin–dentin interface and consists of resin monomers impregnated in the demineralized dentin fibrillar matrix. These resin monomers form resin tags, implementing a micro-mechanical connection between the restoration and the tooth’s dentin [61,62,63]. The hybrid layer may contain a smear layer depending on the adhesive system. In the TE strategy, phosphoric acid rinsing removes the smear layer and exposes the dentinal collagen fibrils, helping them adhere to the adhesive agents. In the SE system, smear layer plugs remain present after applying the SE adhesive, blocking tubular fluids from wetting the surface [11,60,62,63]. The hybrid layer is widely regarded as the weak link of the tooth-restoration interface, as it is susceptible to debonding, biodegradation, and secondary caries initiation [63].

### 6.4. RBC Degradation

#### 6.4.1. Bacterial Degradative Activity towards RBC

Bacterial activity is known to degrade RBC by either acid production [66] or enzymatic activity [67,68,69,70]. Incubating various RBC samples in pH cycles reportedly mimics a bacterial cariogenic acidic challenge, resulting in significantly higher RBC degradation than in neutral pH conditions [66]. *S. mutans* has been found to produce the SMU_118c enzyme, an esterase capable of hydrolysing bisGMA-based and TEDGMA-based RBC [68,70]. This enzyme is highly durable as it can deplete resin composite monomers for a prolonged period in acidic to neutral pH environments, without destabilization in the presence of bisGMA and TEDGMA monomers [68]. Moreover, bishydroxy-propoxyphenyl-propane (bisHPPP), the degradative derivative of bisGMA, has been shown to enhance SMU_118c protein production, resulting in higher bacterial esterase degradative activity towards RBC. This degradative activity, combined with acid production, may compromise the tooth-restoration interface [70].

*E. faecalis* is another bacterium with degradative abilities towards RBC. This bacterium’s esterase-like activity can significantly degrade TE adhesive, followed by RBC and limited degradative activity towards SE adhesive [69]. By producing esterase and collagenolytic activity, this bacterium can weaken the hybrid layer and enable bacterial biofilm penetration into the root canal system through that layer, potentially leading to root canal treatment failure [69]. Both *S. mutans* and *Enterococcus faecalis* contribute to the degradation of the composite restorative materials and the tooth-restoration interface, which results in secondary caries and restoration failure [68,69,70].

#### 6.4.2. Salivary Degradative Activity towards RBC

**Figure 5:**
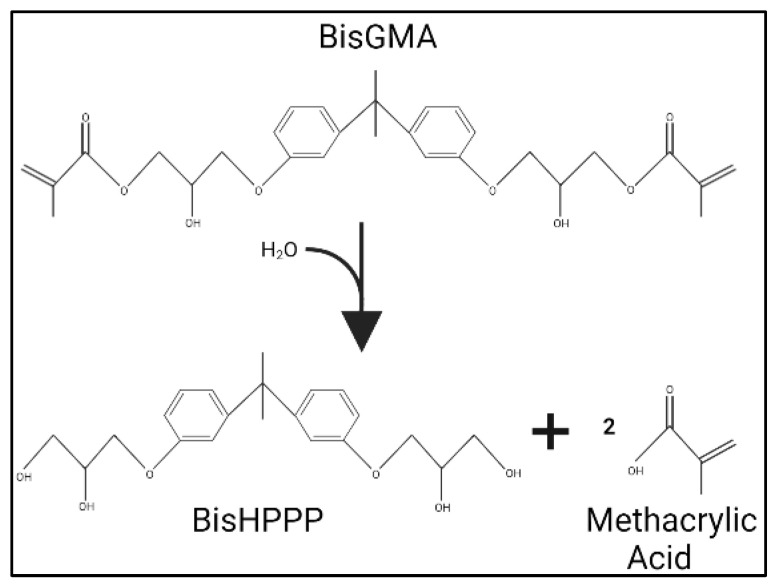
Hydrolysis of bisGMA. Hydrolysis is generated by cleaving the two ester bonds in the presence of water and/or enzymes. The hydrolysis reaction results in the production of bisHPPP and two methacrylic acid molecules.

As previously stated, salivary enzymatic activity can also degrade RBC [14,17] via water penetration as well as salivary enzymatic activity, contributing to the hydrolysis of RBC and the hybrid layer. The presence of water molecules promotes RBC monomer diffusion and ineffective resin polymerization [65], leading to salivary RBC degradation via hydrolysis (Figure 5) [14,15,17]. Human salivary-derived esterase can cleave ester linkage in bisGMA-based composites [17]. The process initially occurs on the outer surface of the RBC, where the salivary enzymes can degrade external ester bonds in the resin matrix and adhesive hybrid layer, leading to decreased interfacial fracture toughness of the restoration. Eventually, the hydrolysing activity slows down depending on the esterase activity’s ability to infiltrate deeper into the RBC [17]. Finer et al. found that Salivary cholesterol esterase (CE)-like and pseudocholinesterase (PCE)-like activity can hydrolyse bisGMA-monomer-containing RBC, resulting in the generation of its degradation product bisHPPP (Figure 5) [14]. Salivary CE- activity and PCE-like activity might act synergistically as they generate higher amounts of RBC degradation than the sum of biodegraded RBC created when they act asynchronously [14]. PCE alone was destabilized by an RBC containing bisGMA/TEGDMA monomers. This effect could be attributed to elevated levels of unreacted monomers and RBC degradation products. However, in the presence of CE, the effect of bisGMA/TEGDMA RBC on PCE was diminished. Another RBC model containing urethane-modified bisGMA/TEGDMA/ethoxylated bisphenol-A dimethacrylate (bisEMA) demonstrated higher biodegradation in the presence of CE and PCE than the amount measured by each enzyme alone [14]. This biodegradative activity, performed by salivary enzymes in conjunction with bacterial enzymes, could result in restoration failure and secondary caries [17].

Degradative salivary activity is not isolated as it may be linked to bacterial activity. The abundant cariogenic bacteria *S. mutans* is up to 0.7 µm in size [71]. Salivary CE- and PCE-like degradative activity promotes marginal gap enlargement. This gap provides microbes access to the exposed tubular collagen type-1 between the RBC and the tooth, allowing further bacterial degradation [29,30,71]. The damaged restoration interface exhibited a high quantity of *S. mutans* biofilm, permitting access to nutrients essential to microbial growth and activity. This finding shows that the coupling nature of salivary degradative activity, paired with bacterial adhesion and dentin demineralization, promotes carious activity, leading to restoration dislodgement and failure [30,70].

Another study by Huang et al. reinforces the above, indicating that the biodegradation of RBC in the presence of salivary esterase activity is a continuous and progressive process resulting in lower marginal stability and fracture toughness [70]. This study also noted that the marginal integrity of RBC increased in the presence of dentinal MMP inhibitors. This implies that preventing harmful collagenase MMP 1 and MMP 8 activity towards demineralized dentinal collagen at the tooth-restoration interface enhanced the restoration’s biostability. This finding further emphasizes that dentinal MMPs play an important role in RBC degradation [70].

#### 6.4.3. Neutrophils Degradative Activity towards RBC

**Figure 6:**
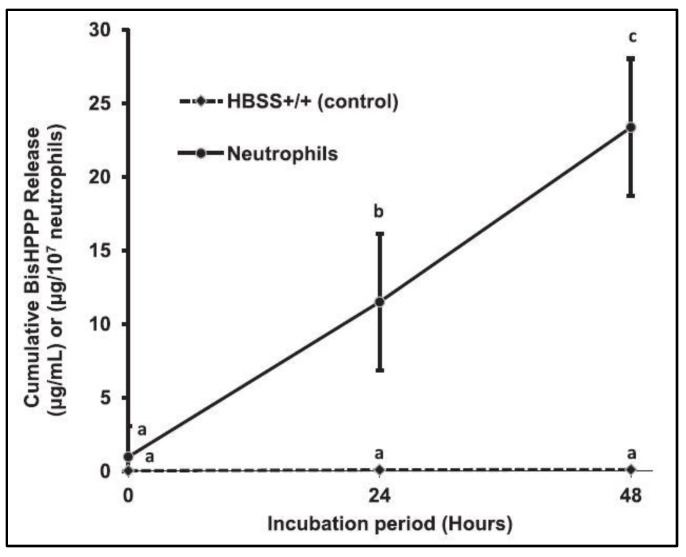
Cumulative bisHPPP release after incubation (37°C) of bisGMA monomers with HBSS alone (control) and with human neutrophils and HBSS. Differences between neutrophil incubation and control incubation can be observed after 24 and 48 h periods (Figure by Gitalis et al., 2020 [19]). Different letters represent statistically different values (*p* < 0.05).

**Figure 7:**
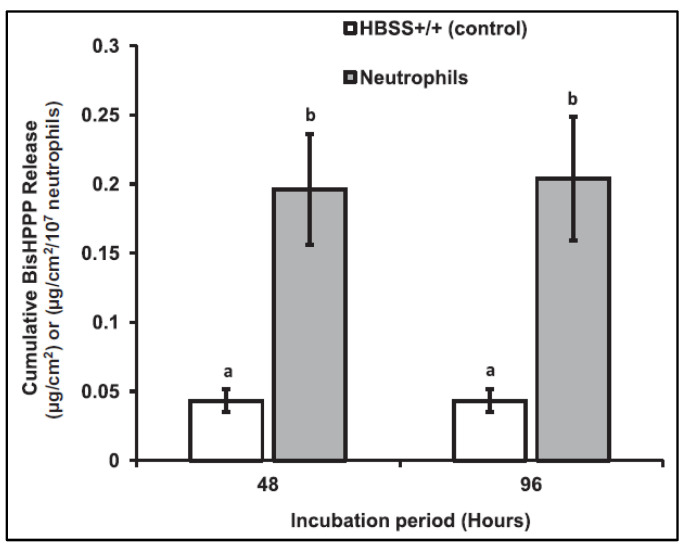
Cumulative bisHPPP release after incubation (37°C) of cured resin composite with HBSS alone (control) and with human neutrophils and HBSS. Differences between neutrophil incubation and control incubation can be observed after 48 and 96 h periods (Figure by Gitalis et al., 2020 [19]). Different letters represent statistically different values (*p* < 0.05).

When incubated with bisGMA monomers (Figure 6), and bisGMA-containing RBC (Figure 7), neutrophils obtain CE-like activities that yield the release of bisHPPP, the degradation derivative of bisGMA, with a reduced bisHPPP release rate after 48 h among the bisGMA-containing RBC [18]. A swift depletion of unreacted or partially reacted bisGMA monomers initiates this degradation on the outer surface of the RBC. The slowdown is attributed to the slower degradation of the cured bisGMA-containing polymer incorporated into the inner portion of the RBC [14,18]. However, in the oral cavity, the masticatory function may accelerate the mechanical deterioration of the RBC, thus exposing more bisGMA monomers to degradation [18,19].

**Figure 8:**
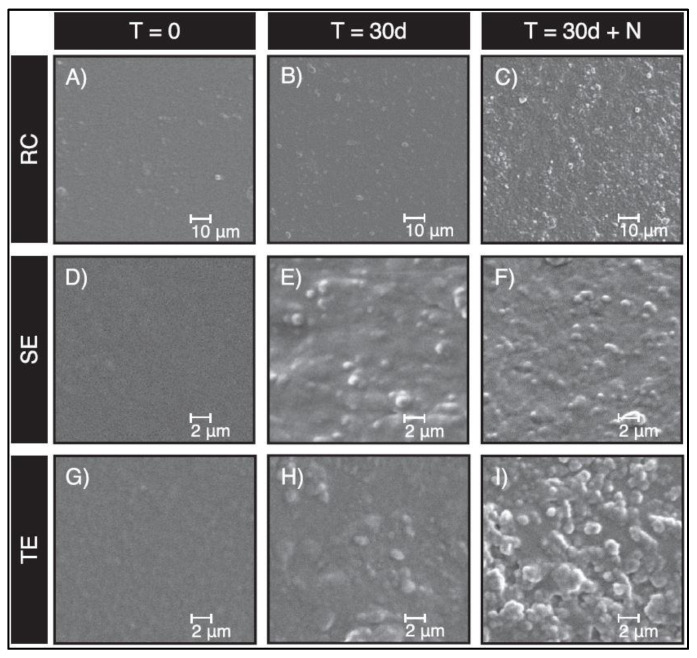
SEM image of resin composite (RBC) (×5000 magnification), self-etch adhesive (SE) (×20,000 magnification), and total-etch adhesive (TE) (×20,000 magnification). Images presented at time of production (T = 0), after 30 days incubation with HBSS saline and LPS (T = 30d), and after 30 days incubation with human neutrophils (N), HBSS saline, and LPS (T = 30d + N). The RBC magnification was lower than SE and TE because the surface properties of RBC could not be observed at ×20,000 magnification due to the high filler load and material roughness (Figure by Gitalis et al., 2020 [19]).

In another study, neutrophils were incubated with RBC, TE, and SE adhesives and stimulants such as Lipopolysaccharides (LPS), Phorbol myristate acetate (PMA), and Formylmethionine-leucyl-phenylalanine (fMLP) [19]. These stimulants were used to promote CE-like activities in neutrophils. LPS has been proven to induce the most abundant CE-like activity as well as pro-inflammatory stimuli and degranulation [19]. Neutrophils reportedly promote RBC and TE adhesive degradation but not SE. The material-dependent difference in degradation could be attributed to the monomer composition of SE, which is more hydrophilic and acidic than TE and RBC [19,70]. Another experiment measured the surface roughness of the incubated samples, which yielded higher surface roughness in RBC and TE adhesives in the presence of neutrophils than in the control. Clinically, restoration surface roughness promotes bacterial adhesion, allowing bacterial accumulation and degradation. Thus, the restoration and the tooth-restoration interfaces are compromised (Figure 8) [19].

Another evaluated mechanical property of the restoration margins was interfacial fracture toughness (FT). Incubation of the specimens with neutrophils reduced FT of TE tooth-restoration interface samples but had a lesser effect on SE samples [19]. This difference could be attributed to the acid-etching process in the TE system, as well as to the hydrophilic and acidic nature of the SE monomers described above. The acid-etching system yields an exposed network of collagen fibrils in demineralized dentin that can be efficiently degraded by neutrophils [19,57,65]. Neutrophils reached a statistically significant FT reduction after 2 and 30 days of incubation, whereas salivary esterases reached a statistically significant FT only after 180 days of incubation [19]. However, the CE-like activity in both salivary esterases and neutrophils is similar, suggesting that the degradative activity towards resin components through CE-like activity should be the same [18,19]. The difference in FT reduction between neutrophils and salivary esterases can be attributed to the collagenolytic activity of neutrophils towards dentin, which enables faster FT reduction combined with their CE-like activity [19].

This study also indicated several enzymes containing resin degradative potential. Cathepsin G, neutrophil elastase, and proteinase 3 are serine proteases. Salivary serine proteases can degrade RBC, and neutrophil proteases, sharing similar properties, are hypothesized to degrade RBC and damage the tooth-restoration interface [14,19]. Myeloperoxidase (MPO) is a peroxidase enzyme produced by neutrophils, which can inflict significant damage by emitting highly reactive oxidants such as hypochlorous acid (HOCL). The esterase CE-like activity of neutrophils promotes the degradation of bisGMA to bisHPPP, may be attributed to MPO and HOCL’s ester hydrolysing abilities [19].

**Figure 9:**
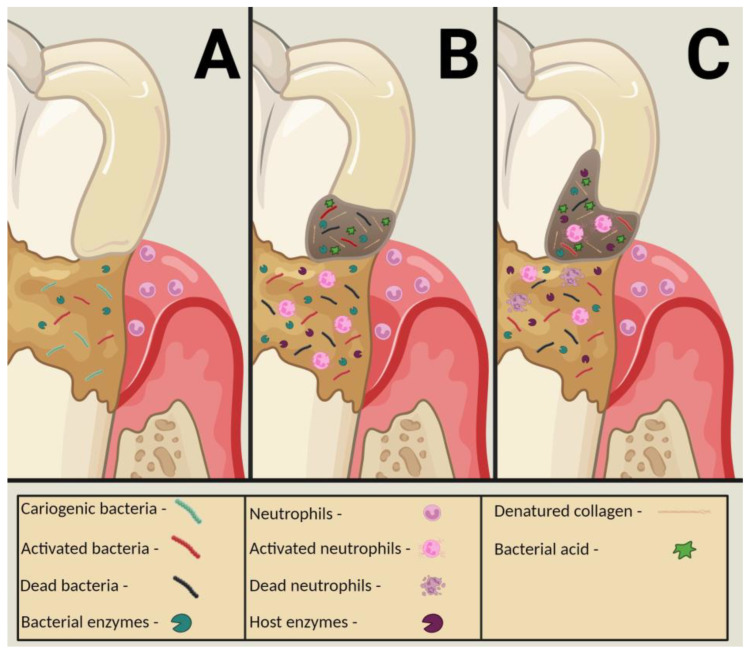
Illustration of a secondary caries lesion. (**A**)—Initial bacterial biofilm colonization of the tooth’s cervical portion near the class II RBC gingival margin, with bacterial enzyme release by activated bacteria. Neutrophils are in the adjacent periodontal sulcus. (**B**)—Advanced bacterial biofilm colonization of the tooth’s cervical portion. Carious cavitation of the gingival tooth-restoration interface with demineralized collagen fibrils initiated by bacterial enzyme release and acid production by activated bacteria, with some bacterial apoptosis. Activated neutrophils migrate to the biofilm and emit neutrophil enzymes. Salivary enzymes are activated. (**C**)—Advanced bacterial biofilm colonization of the tooth’s cervical portion. Advanced carious cavitation at the tooth-restoration interface with lesion progression along the gingival and axial restoration margins. The lesion contains demineralized collagen fibrils initiated by bacterial enzyme release and acid production by activated bacteria, with some bacterial apoptosis. Activated neutrophils migrate to the carious lesion, with some neutrophil apoptosis. Neutrophil, salivary, and dentinal enzymes are activated and promote further degradation of the tooth-restoration interface.

Figure 9 illustrates the mechanism of RBC’s degradation activity for both bacteria and neutrophils. As stated above, the RBC may be initially degraded at the tooth-restoration surface by bacterial acid production [66] and enzymatic activity [67,68,69,70]. In advanced stages, stimulated neutrophils contribute to further degradation and carious lesion progression along the restoration margin [18,19].

## 7. Conclusions

It has been shown that degradative activity derived from saliva, dentin, bacteria, and neutrophils present in the oral cavity can degrade tooth dentin, RBC, resin-based adhesives, and the tooth-restoration interface. Regardless of the source of the proteases, dentinal collagen must be exposed to demineralization prior to host degradation. This step emphasizes the importance of preliminary demineralization either by bacterial activity, dietary acids, or the tooth surface etching process. Neutrophils in the gingival sulcus, adjacent to the root surface and gingival restoration margins, can further degrade tooth-restoration components. This activity, alongside salivary and dentinal degradative activity, weakens the tooth-restoration bond, leading to an expanded marginal gap and reduced bond strength. The extended void can then be colonized by bacterial biofilms, which can further degrade the hybrid layer as well as the exposed demineralized collagen tubules, leading to secondary caries and restoration failure. These important findings greatly implicate the pathogenesis of primary caries, secondary caries, and restoration failure. Future studies should be performed to determine the mechanism by which these endogenous substances function against the tooth-restoration complex.

## Data Availability

Not applicable.
